# Teenage smoking behaviour following a high-school smoking ban in Chile: interrupted time-series analysis

**DOI:** 10.2471/BLT.14.146092

**Published:** 2015-04-29

**Authors:** Andrea B Feigl, Joshua A Salomon, Goodarz Danaei, Eric L Ding, Esteban Calvo

**Affiliations:** a Department of Global Health and Population, Harvard TH Chan School of Public Health, 677 Huntington Ave, 02115 Boston, MA, United States of America (USA).; b School of Business and Economics, Universidad Diego Portales, Santiago, Chile.

## Abstract

**Objective:**

To evaluate the effect of a smoking ban in high schools on smoking behaviour among Chilean students.

**Methods:**

We conducted an interrupted time-series analysis, using repeated cross-sectional data from Chile’s school population survey (2000–2011) for high-school students aged 12–18 years and a control group of persons aged 19–24 years. Poisson regression models were used to assess trends in smoking behaviour before and after the policy changes. The outcome measures were self-reported smoking prevalence (any smoking in the past month) and high frequency of smoking (smoking 15 days or more per month).

**Findings:**

From 2005 to 2011, the prevalence of smoking declined among high-school students by 6.8% per year compared with 3.6% decline per year in the control group. The decline in the target group was 2.9% (95% confidence interval, CI: 0.18 to 5.00) greater. We estimated that 5–6 years after enforcing the law, smoking prevalence among high-school students was 13.7% lower as a result of the ban. The impact of the smoking ban was primarily driven by declines in smoking prevalence among students in grades 8 to 10. The smoking ban did not significantly alter the frequency of smoking.

**Conclusion:**

The 2005 school smoking ban reduced smoking prevalence among younger high-school students in Chile. Further interventions targeting older individuals and frequent smokers may be needed.

## Résumé

**Objectif:**

Évaluer les conséquences d'une interdiction de fumer dans les établissements d'enseignement secondaire sur le comportement tabagique des élèves chiliens.

**Méthodes:**

Nous avons effectué une analyse de séries chronologiques interrompues à l'aide de données transversales répétées provenant d'une enquête sur la population scolaire du Chili (2000-2011) menée auprès d'élèves du secondaire âgés de 12 à 18 ans et d'un groupe de contrôle constitué d'individus âgés de 19 à 24 ans. Des modèles de régression Poisson ont été utilisés pour évaluer l'évolution du comportement tabagique avant et après les changements d'orientation. Les mesures de résultat reposaient sur les auto-déclarations de prévalence du tabagisme (toute consommation de tabac au cours du mois passé) et de fréquence élevée de consommation de tabac (consommation de tabac 15 jours ou plus par mois).

**Résultats:**

De 2005 à 2011, la prévalence du tabagisme a diminué de 6,8 % par an chez les élèves du secondaire; en comparaison, elle a diminué de 3,6 % par an dans le groupe de contrôle. La diminution observée dans le groupe cible était supérieure de 2,9 % (intervalle de confiance, IC, à 95 %: 0,18 à 5,00). Nous avons estimé que 5-6 ans après l'entrée en vigueur de la loi, la prévalence du tabagisme chez les élèves du secondaire avait diminué de 13,7 % grâce à l'interdiction. L'impact de l'interdiction de fumer s'est principalement traduit par une diminution de la prévalence du tabagisme chez les élèves des 8e, 9e et 10e années. L'interdiction de fumer n'a pas considérablement changé la fréquence de consommation de tabac.

**Conclusion:**

L'interdiction de fumer dans les écoles, qui date de 2005, a diminué la prévalence du tabagisme chez les plus jeunes élèves des établissements d'enseignement secondaire du Chili. D'autres interventions ciblant des individus plus âgés et des fumeurs fréquents pourraient être nécessaires.

## Resumen

**Objetivo:**

Evaluar el efecto de la prohibición de fumar en los institutos sobre el consumo de tabaco entre los alumnos chilenos.

**Métodos:**

Se llevó a cabo un análisis de series temporales interrumpidas utilizando datos transversales repetidos obtenidos de la encuesta de la población escolar de Chile (2000-2011) en alumnos de instituto de entre 12 y 18 años y con un grupo de control de personas de entre 19 y 24 años. Se utilizaron modelos de regresión de Poisson para evaluar las tendencias en el consumo de tabaco antes y después de los cambios en la política. Los indicadores de resultados eran la prevalencia autodeclarada del consumo de tabaco (cualquier consumo de tabaco en el último mes) y la alta frecuencia del consumo de tabaco (fumar 15 o más días al mes).

**Resultados:**

De 2005 a 2011, la prevalencia del consumo de tabaco se redujo en un 6,8% anual entre los estudiantes de instituto, en comparación con la reducción del 3,6% anual en el grupo de control. El descenso en el grupo objetivo fue de un 2,9% (intervalo de confianza, IC, del 95%: de 0,18 a 5,00) superior. Se estimó que 5-6 años después de la aplicación de la ley, la prevalencia del consumo de tabaco entre los alumnos de instituto era un 13,7% menor como consecuencia de la prohibición. El impacto de la prohibición de fumar se notó principalmente en la disminución del consumo de tabaco entre los alumnos de instituto de octavo a décimo grado. La prohibición de fumar no alteró significativamente la frecuencia del consumo de tabaco.

**Conclusión:**

La prohibición de fumar en las escuelas de 2005 redujo la prevalencia del consumo de tabaco entre los alumnos de instituto más jóvenes en Chile. Es posible que sean necesarias intervenciones adicionales dirigidas a individuos de más edad y a fumadores frecuentes.

## ملخص

**الغرض:**

تقييم تأثير حظر التدخين في المدارس الثانوية في سلوك التدخين بين الطلاب في شيلي.

**الطريقة:**

لقد أجرينا تحليل سلاسل زمنية متقطِّعة، وذلك باستخدام بيانات مقطعية عرضية متكررة من مسح جمهور الطلاب في المدرسة في شيلي (في الفترة ما بين عام 2000 إلى عام 2011) لطلاب المدارس الثانوية الذين تتراوح أعمارهم ما بين 12 إلى 18 عامًا ومجموعة الشاهد من الأشخاص الذين تتراوح أعمارهم ما بين 19 إلى 24 عامًا. واستخدمت نماذج التحوف لبواسون لتقييم النزعات في سلوك التدخين قبل وبعد تغيرات السياسة. كانت قياسات الحصائل عبارة عن معدّل انتشار التدخين المبلّغ عنه ذاتيًا (أي حالة تدخين في الشهر الماضي) وارتفاع وتيرة التدخين (التدخين لمدة 15 يومًا أو أكثر في الشهر).

**النتائج:**

في الفترة من عام 2005 إلى عام 2011، انخفض معدل انتشار التدخين بين طلاب المدارس الثانوية بنسبة 6.8 % سنويًا مقارنة مع انخفاض بنسبة 3.6 % سنويًا في مجموعة الشاهد. كان الانخفاض في المجموعة المستهدفة بنسبة 2.9 % (بنسبة أرجحية مقدارها 95 %: 0.18 إلى 5.00) أكبر. ولقد قدرنا أنه بعد تطبيق القانون لمدة من 5 إلى 6 أعوام، كان معدل انتشار التدخين بين طلاب المدارس الثانوية أقل بنسبة 13.7 % كنتيجة لهذا الحظر. ونتج تأثير حظر التدخين بشكل رئيسي عن الانخفاض في معدلات انتشار التدخين بين الطلاب في الصفوف من 8 إلى 10. ولم يغير حظر التدخين كثيرًا من وتيرة التدخين.

**الاستنتاج:**

أدى حظر التدخين في المدارس في عام 2005 إلى انخفاض معدل انتشار التدخين بين طلاب المدارس الثانوية الأصغر في شيلي. قد تكون هناك حاجة إلى إجراء المزيد من التدخلات التي تستهدف الأفراد الأكبر سنًا والمدخنين بشكل متكرر.

## 摘要

**目的:**

旨在评估高中禁烟令对智利学生吸烟行为的影响。

**方法:**

我们开展了一项断续时间序列分析，所采用的重复性横断面数据来源于智利学校人口调查（2000–2011 年），其中的调查对象为年龄介于 12 至 18 岁的高中学生，同时以年龄介于 19 至 24 岁的人群作为对照组。 泊松回归模型用于评估吸烟行为在政策改变之前和之后的趋势。 效果衡量指标为自报吸烟流行率（在过去一个月中的任何吸烟行为）以及频繁程度高的吸烟行为（每个月吸烟 15 天或更多）。

**结果:**

从 2005 到 2011 年，高中学生的吸烟流行率每年下降 6.8%，而对照组每年下降 3.6%。 目标群体的下降率为 2.9%（95% 置信区间，CI：0.18 至 5.00）以上。 我们估计在强制执行法律后的 5 至 6 年内，高中学生的吸烟流行率会因该禁令而降低 13.7%。 禁烟令的影响主要是由于吸烟流行率在 8 至 10 年级的学生中下降而引起的。禁烟令并没有显著改变吸烟的频繁程度。

**结论:**

2005 年颁布的学校禁烟令使智利境内较为年轻的高中学生降低了吸烟流行率。我们可能需要针对较为年长的个人和吸烟频繁者实施进一步的干预措施。

## Резюме

**Цель:**

Оценить, каким образом запрет на курение в старших классах чилийских школ отразился на курении учеников.

**Методы:**

Был проведен анализ статистических данных, полученных в результате многократных опросов старшеклассников (12–18 лет) и контрольной группы взрослых (19–24 года) в Чили в 2000–2011 гг., методом прерванного временного ряда. Для оценки поведенческих тенденций в отношении курения до изменения государственной политики и после этого применялись регрессионные модели Пуассона. В качестве критериев оценки использовались личные показания опрошенных о самом факте курения (курили ли опрошенные в течение прошлого месяца) и о высокой частоте курения (курение в течение как минимум 15 дней в прошлом месяце).

**Результаты:**

В период с 2005 по 2011 год распространенность курения среди школьников старших классов каждый год снижалась на 6,8%. В контрольной группе снижение составило 3,6%. В целевой группе снижение было выше на 2,9% (95% доверительный интервал, ДИ: от 0,18 до 5,00). По нашим оценкам, спустя 5–6 лет после введения закона в действие распространенность курения среди старшеклассников в результате запрета снизилась на 13,7%. Действие запрета на курение в основном проявилось в том, что стали меньше курить школьники 8–10 классов. Запрет на курение не оказал существенного влияния на частоту курения.

**Вывод:**

Запрет на курение в школах в 2005 году снизил распространенность курения среди чилийских старшеклассников младшего возраста. Для более взрослых и часто курящих лиц могут потребоваться дополнительные интервенционные меры.

## Introduction

The detrimental health effects of tobacco smoking and use are widely documented.^1^^–^^7^ Every year, first-hand smoking causes five million deaths globally^8^ and 80% of smoking-related deaths occur in low- and middle-income countries.^8^^,^^9^ To reduce such mortality and morbidity, policy-makers can enforce policies such as public smoking bans,^10^ subsidized cessation programmes,^11^^–^^13^ tobacco warning labels^14^^–^^16^ and tax increases.^17^^–^^19^ However, the effect of these legislative changes differ based on context – compliance with smoking bans might be higher in countries with lower smoking prevalence.^20^

There is a high prevalence of tobacco smoking in Chile, with 44% of adult males and 38% of adult females smoking in 2011, which was the highest prevalence in Latin America at that time.^21^ In 2009, Chile also had the second highest teenage smoking prevalence based on the Global Youth Tobacco Survey, with an annual smoking prevalence of 34.2%.^9^ The Chilean government ratified the WHO Framework Convention on Tobacco Control in 2005^22^ and passed tobacco legislation that took effect on 1 January 2006.^23^

One provision in the legislation is a smoking ban, including a cigarette sales ban, within 300 metres of all high schools, which was the only provision that was enforced with 100% reported compliance (further information available from author). Preliminary evidence suggests that between 2005 and 2011, there was a decline in smoking prevalence among high-school students, but not in the general adult population.^24^ Here we assess changes in smoking prevalence among high-school students before and after the smoking ban.

## Methods

### Study design

We used interrupted time-series to evaluate trends in smoking prevalence before and after the smoking ban in the target population (high-school students aged 12–18 years) and compared it to the general population aged 19–24 years, which we assumed to be unaffected by the ban. We assumed a similar institutional environment in both groups, since in Chile, over 40% of individuals aged 19–24 years attended a university or a secondary institution in 2008.^25^ To ensure that the control group did not include anyone subjected to the ban, the control population in 2000/2001 to 2006/2007 included individuals aged 19–24 years; in 2008/2009 individuals aged 20–24 years; and in 2010/2011 individuals aged 22–24 years.

### Data

For the target population, we used data from the school population survey – a biennial and regionally representative survey on substance abuse and addictive behaviour in the Chilean school population, including the last grade of primary school (8th grade, mean age 13.5 years) and the high-school population (9th to 12th grade, ages 14–18 years). The school surveys were conducted in odd years; data from 2001–2011 were used in the analyses.^26^ The school population survey employed a stratified, probabilistic two-stage sampling procedure.

For the control population, we used data from the general population survey,^26^ conducted in even years, for 2000–2010. We combined data from the school and the general population surveys for before (2000/2001, 2002/2003 and 2004/2005) and after (2006/2007, 2008/2009 and 2010/2011) the implementation of the law. For each biennium, we analysed an average sample size of 50 000 individuals in the target group and 2300 individuals in the control group (further details available from the author).

### Statistical analyses

To compare trends in smoking prevalence, we used self-reported, past 30-day smoking prevalence (yes/no) as the primary outcome. As a secondary outcome, we created a frequent smoking variable indicating smoking 15 or more days per month during the last month (yes/no).

To model the outcomes, we used Poisson regression models to estimate prevalence ratios.^9^^,^^27^^–^^29^ The models were run separately for control and target groups due to different variances arising from different samples and sample frames. In multivariable regression models, we controlled for age, sex and region and included an interaction term for age and sex. For the target group we also controlled for school type, grade-level and grade point average, and in the control group for socioeconomic status and method of survey administration. Absence of data in covariates was generally lower than 2% and the complete case method was chosen for the main analysis. We then conducted a two-sample *t*-test with unequal variances conducted on the estimated coefficient for post-intervention change in the target group compared to the coefficient in the control group. All analyses were performed using Stata version 12 (StataCorp LP. College Station, United States of America).

We used a 2-stage model with time-dependent spline term for trend before and after 2005 (Table 1). At stage 1, we ran separate robust Poisson regression models for each intervention and control group, specified as follows:
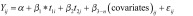
(1)where *Y* represents the binary smoking variable of interest, for individual *i* at time *j*; *ε*represents standard errors clustered at the municipal level (high schools) and regional level (universities) and *t* represents the time period. In the first stage, the models (model a and model b) were run separately for each group.

**Table 1:** Values for time period variable in first stage Poisson regressions to estimate trends in tobacco smoking, Chile, 2000–2011

Time period	Survey period					
	2000/2001	2002/2003	2004/2005	2006/2007	2008/2009	2010/2011
**1**	1	3	5	7	9	11
**2**	0	0	0	2	4	6

At stage 2, we were interested in the *β*_2_ coefficients of each model, reflecting the annual rate of change in smoking prevalence during the post-intervention period, relative to the annual rate of change in the pre-intervention period. The first null hypothesis (H_0_1) that was tested in the analyses was:
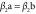
(2)the second null hypothesis (H_0_2) was:
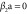
(3)the third null hypothesis (H_0_3) was:


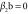
(4)

H_0_1 was tested via a two-sample *t*-test with unequal variances, and H_0_2 and H_0_3 were tested via the significance test of the coefficient in each model.

We conducted several sensitivity analyses: (i) including all aged 19–24 years in the control population; (ii) running analogous analyses using past 30-day cannabis use as the main outcome (to test for the specificity of the school smoking ban on smoking behaviour versus addictive behaviour in general); (iii) including only those municipalities measured in every survey; (iv) excluding individuals with less than high-school education in the control group; (v) including additional control variables (alcohol consumption, and religion); (vi) including additional control variables (alcohol consumption, religion, and paternal education); (vii) adjusting for survey weights; (viii) adjusting for complex survey design; (ix) using the general population survey for individuals aged 19–64 years as the control population; and (x) using the missing indicator method to account for missing data.^30^ For smoking frequency, we conducted the two sensitivity analyses – adjusting for complex survey design and stratifying the analysis by grade level in the target group – to investigate the robustness of the result.

### Ethics approval

The study was exempt from ethics approval as it only used secondary data from surveys that had followed conventional ethics guidelines and recorded no personal identifiers.

## Results

We obtained data on 319 798 individuals surveyed between 2000 and 2011. Characteristics for the target and control groups are presented in Table 2. Unadjusted for covariates, 30-day smoking prevalence among high-school students was highest in 2000/2001, with a prevalence of 41.9% (23 822/56 817) and slightly declining to 40.1% (23 678/59 101) in 2004/2005. After the implementation of the law, the prevalence declined to 25.7% (8596/33 509) in 2010/2011. Smoking prevalence in the control group was higher overall, with the highest in 2002/2003 (58.2%; 1132/1945). Before the law came into effect the prevalence was 57.3% (1104/1927) which then steadily declined to 44.9% (405/902) by 2010/2011. Immediately after 2005, there was a greater decline in smoking prevalence among high-school students than among the control population (40.1% to 34.9% and 57.3% to 54.8%, respectively).

**Table 2:** Characteristics of study populations, Chile, 2000–2011

Characteristic	High-school population aged 12–18 years							Control population aged 19–24 years,^a^					
	2000/2001	2002/2003	2004/2005	2006/2007	2008/2009	2010/2011		2000/2001	2002/2003	2004/2005	2006/2007	2008/2009	2010/2011
	*n* = 56 817	*n* = 57 032	*n* = 59 101	*n* = 51 432	*n* = 48 213	*n* = 33 509		*n* = 5 466	*n* = 1 945	*n* = 1 927	*n* = 1 937	*n* = 1 517	*n* = 902
Age, years (SD)	15.5 (1.5)	15.6 (1.5)	15.2 (1.5)	15.4 (1.5)	15.4 (1.5)	15.5 (1.5)		21.5 (1.7)	21.6 (1.7)	21.6 (1.7)	21.5 (1.7)	22.0 (1.43)	23.0 (0.80)
Number of men, (%)	27 953 (49.2)	28 117 (49.3)	29 019 (49.1)	25 458 (49.5)	23 673 (49.1)	16 520 (49.3)		2 603 (47.6)	1 093 (56.2)	1 045 (54.2)	1 038 (53.6)	712 (46.9)	409 (45.3)
Numbers of smokers in the past 30 days, (%)	23 822 (41.9)	22 194 (38.9)	23 678 (40.1)	17 950 (34.9)	15 910 (33.0)	8 596 (25.7)		3 012(55.1)	1 132 (58.2)	1 104 (57.3)	1 061 (54.8)	769 (50.7)	405 (44.9)
Number of days smoked per month	NA	16.5	14.1	15.0	14.5	13.0		NA	21.3	21.4	19.8	20.9	19.9
Number of high frequency smokers,^b^ (%)	NA	11 076 (19.4)	9 675 (16.4)	7 951 (15.5)	6 928 (14.4)	3 364 (10.0)		NA	832 (42.8)	809 (42.0)	724 (37.4)	529 (34.9)	280 (31.0)
Number of cannabis users in past 30 days, (%)	4 432 (7.8)	3 992 (7.0)	3 605 (6.1)	4 269 (8.3)	3 857 (8.0)	3 250 (9.7)		364 (6.7)	134 (6.9)	137 (7.1)	140 (7.2)	119 (7.8)	56 (6.2)

NA: not applicable; SD: standard deviation.

^a^ The control population in 2000/2001 to 2006/2007 includes individuals aged 19–24 years, in 2008/2009 individuals aged 20–24 years; and in 2010/2011 individuals aged 22–24 years.

^b^ Smoking more than 15 days per month.

Smoking frequency was higher in the control group than in the target group. In 2002/2003, high-school individuals smoked an average of 16.5 days per month, which declined to 15.0 days per month in 2006/2007 and further dropped to 13.0 days per month in 2010/2011. The number of days smoked per month in the control group was relatively stable between 2000 and 2011, with a peak of 21.4 days during 2004/2005 and by 2010/2011 this had declined to 19.9 days. In all groups and grades, average smoking frequency was highest in 2002/2003 and lowest in 2010/2011. Smoking prevalence and frequency increased with increasing school grade (data available from author).

### Smoking prevalence

Adjusting for covariates in the multivariable regression model, smoking prevalence significantly increased among high-school students over the period 2000–2005, from an adjusted smoking prevalence of 37.4% in 2000/2001, to an adjusted smoking prevalence of 38.6% in 2004/05 (*P* = 0.014); there was no significant trend observed in the control group. After 2005, there was a significant decline in smoking prevalence in the target group (*P* < 0.001), resulting in an adjusted smoking prevalence of 24.9% in 2010/11 in the high-school population. The prevalence ratio of the smoking prevalence change before versus after the law in the control versus the target group was 0.971 annually (95% confidence interval, CI: 0.950 to 0.992), or 0.863 (95% CI: 0.774 to 0.961) over the course of the intervention (Table 3). Thus, smoking prevalence after 2005 declined significantly faster in the target group than in the control group. 

**Table 3:** Changes in smoking prevalence before and after the implementation of a high-school smoking ban in Chile, 2000–2011

Policy period	Annual prevalence change, PR^a^ (95% CI)			
	Target group^b^	Control group^c^	Intervention effect^d^	
			Per year	Five years
Pre-policy (2000–2005)	1.006 (1.001 to 1.012)	1.010 (0.999 to 1.02)	0.971 (0.950 to 0.992)	0.863 (0.774 to 0.961)
Post-policy (2006–2011)	0.932 (0.927 to 0.937)	0.964 (0.953 to 0.974)		
Difference between pre-policy and post-policy	0.926 (0.917 to 0.934)	0.953 (0.935 to 0.973)		

CI: confidence interval; PR: prevalence rate ratio.

^a^ PRs represent the annual decrease in smoking prevalence.

b High-school population aged 12–18 years. Adjusted for age, sex, region, school-type, course, and sex * age.

c Aged 19–24 years. Adjusted for age, sex, region, socioeconomic status, survey method, and sex * age.

^d^ Difference in changes of smoking prevalence between target and control group.

Since the descriptive analysis revealed a difference in smoking prevalence among different high-school grades (data available from author), a grade-based stratified analysis was done to determine the grades in which the law had the greatest effect. The smoking ban was most effective among students in the lowest grade, leading to a 7.2% (95% CI: 4.3 to 10.1) annual improvement in smoking trend in this population versus the control group. The ban was least effective among students in the two highest grades, where no significant effect on smoking prevalence was detected (Table 4).

**Table 4:** Changes in smoking prevalence before and after the implementation of a high-school smoking ban in Chile, 2000–2011 by school grade

Policy period	Annual prevalence change, PR^a^ (95% CI)					
	Target group^b^ by school grade					Control group^c^
	8th	9th	10th	11th	12th	
Pre-policy (2000–2005)	1.02 (1.001 to 1.03)	1.01 (0.999 to 1.02)	1.01 (0.999 to 1.02)	0.998 (0.990 to 1.01)	0.995 (0.941 to 1.00)	1.010 (0.999 to 1.02)
Post-policy (2006–2011)	0.898 (0.886 to 0.910)	0.930 (0.923 to 0.938)	0.930 (0.923 to 0.937)	0.940 (0.934 to 0.947)	0.948 (0.941 to 0.956)	0.964 (0.953 to 0.974)
Difference between pre-policy and post-policy	0.885 (0.864 to 0.906)	0.922 (0.908 to 0.937)	0.923 (0.911 to 0.936)	0.942 (0.930 to 0.955)	0.942 (0.930 to 0.955)	0.953 (0.935 to 0.973)
Intervention effect^d^	0.928 (0.899 to 0.957)	0.967 (0.943 to 0.992)	0.968 (0.945 to 0.992)	0.988 (0.965 to 1.01)	1.00 (0.975 to 1.02)	NA

CI: confidence interval; NA: not applicable; PR: prevalence rate ratio.

^a^ PRs represent the annual decrease in smoking prevalence.

^b^ High-school population aged 12–18 years. Adjusted for age, sex, region, school-type, course, and sex * age.

c Aged 19–24 years. Adjusted for age, sex, region, socioeconomic status, survey method, and sex * age.

### Smoking frequency

Before the ban, frequent smoking in the target group declined by 2.5% annually from 2000 to 2005 (95% CI: −4.1 to −0.8); while in the control group no significant trend was observed. After 2005, the difference in smoking frequency between the target and control group was not significant (*P* = 0.58; Table 5).

**Table 5:** Change in prevalence of high frequency smoking^a^ before and after the implementation of a high-school smoking ban in Chile, 2000–2011

Policy period	Annual prevalence change, PR^b^ (95% CI)		
	Target group^c^	Control group^d^	Intervention effect^e^
Pre-policy (2000–2005)	0.975 (0.959 to 0.992)	0.992 (0.959 to 1.03)	0.986 (0.938 to 1.036)
Post-policy (2006–2011)	0.924 (0.916 to 0.932)	0.953 (0.938 to 0.969)	
Difference between pre-policy and post-policy	0.947 (0.926 to 0.969)	0.961 (0.919 to 1.00)	

CI: confidence interval; PR: prevalence rate ratio.

^a^ Smoking more than 15 days per month.

^b^ PRs represent the annual decrease in smoking prevalence.

c High-school population aged 12–18 years. Adjusted for age, sex, region, school-type, course, and sex * age.

d Aged 19–24 years. Adjusted for age, sex, region, socioeconomic status, survey method, and sex * age.

^e^ Difference in changes of smoking prevalence between target and control group.

### Sensitivity analyses

When examining the changes in cannabis use before and after the implementation of the law, after 2005 cannabis use increased significantly in the target group, but declined in the control population. Hence, after 2005, past 30-day cannabis use increased by a 14% (95% CI: 11 to 18) greater rate in the target population compared to the control population (data available from author).

Restricting the analysis to municipalities that were included in all survey years yielded identical effect size estimates as the main analysis (data available from author). When including only those with a high-school diploma in the control population, the effect size for the smoking ban increased to 4.5% (95% CI: 1.3 to 7.5). Adjusting for alcohol consumption, religion and paternal education in the target group also increased the effect size estimate to 4.8% (95% CI: 2.4 to 6.4). The results for smoking frequency were robust to inclusion of complex survey design and educational grade (data available from author).

## Discussion

Here we demonstrate that smoke-free zones and tobacco sales restrictions in high schools can be effective in the Chilean context. We observed a greater decline in smoking prevalence in the target group than in the control group after the implementation of the law. The average intervention effect was driven by the decline in smoking prevalence among younger high-school students. In contrast, the smoking ban proved ineffective in lowering prevalence among older students, as well as in reducing the frequency of smoking. These results suggest that the smoking ban prevented smoking initiation and selectively targeted low-frequency smokers. These findings are consistent with the theory that non-smokers and less frequent smokers are most receptive to tobacco-control policies.^31^

The similar decline in frequent smoking in both groups suggests that the ban did not affect how often people smoke and therefore better-targeted programmes and policies are needed for frequent smokers. Examples of effective programmes include smoking-cessation counselling in high schools and free prescription of nicotine patches.^11^ Legislation featuring such programmes has been proposed, but not yet passed or funded in Chile.

Several sensitivity analyses supported the robustness of our results. When we included all individuals aged 19–24 years in the control group, we found that the effect estimate was lower since the control group in 2008 and 2010 also included people previously targeted by the law. In contrast, when including only those with a high-school diploma in the control group, the effect size increased. A possible explanation is that those with at least a high-school diploma continued, rather than altered, their smoking behaviour between 19 and 24 years of age. Applying cannabis use as the main outcome suggested that relative to the control group, cannabis use in high-school students increased after the implementation of the law. This is further evidence that the decline in smoking prevalence among high-school students is attributable to the smoking ban. Our findings confirm results from previous studies that increased difficulty of obtaining cigarettes was associated with less positive views about smoking among adolescents^32^ and lower smoking prevalence among Chilean teenagers.^33^

In addition to the immediate effect of the law, the long-term effect on disease burden should also be considered. Emerging evidence suggests that adolescence is the most critical period in life for development of long-term addictive behaviours.^34^^–^^36^ In the USA, it has been shown that laws on youth access to tobacco might have long-term effects on smoking prevalence, however, these effects might be limited to women only.^37^ To estimate whether the smoking ban would have a long-term effect on morbidity, one can use a life table approach such as the 1960–1972 American cohort. In this cohort, the mortality rate in men aged 35–39 years, who have never smoked regularly, was 1.34 per 1000 person-years, versus 2.55 in those who smoked one or more packs of cigarettes per day.^38^ In the age group 40–44 years, mortality in men who never smoked was 1.93 per 1000 person-years, versus 4.59 in frequent smokers (smoking more than 20 cigarettes per day).^38^ Overall mortality rates in women were lower in both smokers and non-smokers, but still substantially higher in frequent smokers than non-smokers. Assuming that the mortality rates from this American cohort apply to the current Chilean high-school population and that the effect of the ban on prevalence persists long-term, the Chilean high-school smoking ban could potentially reduce mortality by more than twofold 20 years from now. This reduction would result in better health outcomes for a large number of people as well as reducing health expenditure on smoking-related diseases like lung cancer or chronic obstructive pulmonary disease.^39^^–^^43^ However, there is uncertainty as to how long this effect on smoking behaviour will last, and long-term assessments are needed.

This study has limitations. First, individuals were not randomly assigned to the target group and control group. However, all provisions of the 2005 law uniformly affected Chile’s general population (data available from author), except for the high-school smoking ban. Thus, even if the law affected the control group, the same section of the law also affected the target group. Second, the average age of smoking initiation was 13.2 years in Chile and older individuals are less likely to start or quit smoking. Hence, individuals in the control group (19–24 years) – both smokers and non-smokers – may have a lower propensity to change their behaviour in response to a similar ban. This could have led to an upward bias in the effect size estimates. However, in a sensitivity analysis using the general population aged 25–64 years as a control group, the effect sizes were not significantly higher. Third, the cross sectional design of our study means that we cannot identify specific outcomes of the ban at an individual level. We cannot distinguish, for example, whether the ban prevented people from starting smoking or encouraged current smokers to stop. Neither can we estimate the effect of the ban on overall levels of tobacco consumption per capita. Fourth, there were no data on how schools enforced the ban. Additional data on compliance with the smoking ban at Chilean high schools – similar to a study conducted in India^44^ – could have further strengthened our conclusions.

Smoking prevalence is often highly correlated with socioeconomic status.^45^ In our study, socioeconomic status variables in both target and control group were highly collinear with neighbourhood. For this reason, socioeconomic status variables were omitted from the main analyses. Future studies could explore equity concerns relating to the high-school smoking ban.

In conclusion, smoking bans in schools can be effective in reducing the number of teenagers smoking but not in decreasing the frequency of smoking. These are relevant lessons for both national and global policy-makers. Chile’s 2013 tobacco control legislation extended the smoking bans to include bars, restaurants and universities, which may help to further decrease smoking prevalence. However, both the 2005 and 2013 tobacco legislation offered little help to those in need of cessation counselling. Future policies should include accessible cessation programmes, funds for enforcing smoke-free public spaces and other provisions to help people stop smoking.
